# Supplementary material for: Trends of attrition from HIV care and its predictors among adolescent girls and young women with inconsistent viral load suppression results in Mainland Tanzania, 2016–2024
Source: PLOS Glob Public Health. 2026 Feb 11;6(2):e0005489. doi: 10.1371/journal.pgph.0005489 (PMC12893582; doi:10.1371/journal.pgph.0005489)
Supplement: S1 Table — (DOCX) [file pgph.0005489.s001.docx]

**S1 Table. Categorization and coding of independent variables.**

| **Variable** | **Definition** | **Coding** |
| --- | --- | --- |
| Age at ART initiation | Age calculated from date of birth to date of ART start | 15–19 years, 20–24 years |
| Age at study enrollment | Age of the client at the time of study enrollment | 0–14 years, 15–19 years, 20–24 years |
| Residence | Geographical location of the client | Urban, Rural |
| Marital status | Self-reported marital status from CTC2 database | Single, Married, Divorced/Separated |
| Type/Level of health facility | Healthcare facility hierarchy based on service complexity | Dispensary, Health Center, Hospital |
| Health facility ownership | Ownership and management type of healthcare facility | Government, Private, Faith-Based Organization |
| Zone | Zone of Mainland Tanzania based on geographical regions | Coastal, Northern Highland, Lake, Central, Southern Highland, Western |
| Entry point at HIV diagnosis | Location/service point where HIV diagnosis was made | OPD/IPD, RCH/PMTCT, VCT, Community, Others |
| Initial viral load | First viral load after 6 months of ART, in copies/mL | <50 (Undetectable), 50–999 (LLV), ≥1000 (Unsuppressed) |
| Baseline CD4+ cell count | CD4 count measured at ART initiation using flow cytometry | <200, 200–500, >500 cells/mL |
| Baseline HIV disease stage | WHO clinical staging (I–IV) at ART enrollment | Stage I, II, III, IV |
| Duration on ART | Time on ART from initiation to study endpoint | <4 years, ≥4 years |
