# Supplementary material for: Trends of attrition from HIV care and its predictors among adolescent girls and young women with inconsistent viral load suppression results in Mainland Tanzania, 2016–2024
Source: PLOS Glob Public Health. 2026 Feb 11;6(2):e0005489. doi: 10.1371/journal.pgph.0005489 (PMC12893582; doi:10.1371/journal.pgph.0005489)
Supplement: Attachment — Submitted filename: PONE Review JR 01122025.docx [file pgph.0005489.s002.docx]

**PONE Review: PGPH-D-25-03362**

**Title:** Trends of attrition from HIV care and its predictors among Adolescent Girls and Young Women with inconsistent viral load suppression results in Mainland Tanzania, 2016–2024

**Introduction**

The study presented is highly relevant, addressing a critical gap in understanding attrition from HIV care among adolescent girls and young women (AGWY) in Tanzania Mainland. AGWY is a population disproportionately affected by HIV in Tanzania. Therefore, by leveraging national routine program data over eight years’ period, the authors provide valuable insights into the patterns and determinants of loss from care among a population that is central to achieving national HIV targets. The findings have important implications for improving programmatic responses and guiding targeted interventions. My detailed comments are outlined below.

| **Section** | **Comment** | **Line number** | **Suggestion** |
| --- | --- | --- | --- |
| Abstract |  |  |  |
| Introduction | Please revise the following sentence, it has a long phrasing.  Alarmingly, 3,100 60 of these new infections occur in Sub-Saharan Africa (SSA), accounting for 77% of all new AGYW 61 infections globally in 2023, with Eastern and Southern Africa alone contributing around 60%. | 59-61 | Revise to enhance readability |
|  | Do the authors mean the UNAIDS 95 targets were set in response to new HIV infections in AGWY?  Starting the sentence with the words ‘’in response… links UNAIDS 95 targets to the preceding problem of high HIV infections among AGYW. The authors need to revise for clarity. | 62 |  |
|  | What about treatment as prevention as a strategy to reduce new HIV infections by ensuring that PLHIV who achieve and maintain viral suppression (undetectable viral load) cannot transmit the virus.  Lines 69-70 touches about ART coverage rather than a prevention strategy. | 67-68 | Revise these sentences to include treatment as prevention strategy to reduce new infections/ HIV transmission. |
|  | Kindly change the word **patients** to use person first language to avoid stigmatization. Please use PLHIV or recipients of care. | 74 | Use person first language. |
|  | Please clarify who are these recipients of care described here with retention challenge? | 73-79 |  |
|  | Is the data described from Tanzania? Given that the authors have started a new paragraph describing data of viral suppression among AGWY. | 85-88 |  |
|  | Replace the word **exploring** with **examined**. Note that the study applied quantitative approaches measuring trends and associations. | 93-94 | Use past tense here.  This study **examined** trends in ART care attrition, survival time in care, and associated factors among AGYW in Tanzania Mainland. |
| **Methods** |  |  |  |
|  | Replace **“data of”** with **“data from people living with HIV”** | 97 |  |
|  | Healthcare providers at over 1,100 facilities may sound overstating. | 99 | Please specify the number of health facilities and, specify if they are public and private. |
|  | Inconsistent viral load suppression is vague a reader may have several interpretations. Also, the authors did not state what time period was allowed between the two viral load results? Were the viral load results sequential? | 102 | Either define what you mean by inconsistent viral load suppression or maintain the definition of non-viral suppression in lines 102-103.  **Note that two viral load results ≥1000 copies/ml at least six months after ART initiation is non-viral suppression or ART virological failure.** |
|  | Please clarify whether 2016–2024 refers to timeframe for inclusion of study participants’ initial visits and follow up. |  |  |
|  | It is not clear if participants enrolled between 2016 and 2019 means those enrolled in HIV care or those initiated on ART. | 103 | Please clarify what enrolled between 2016 and 2019 means. |
|  | The sentence here may be clearer if the same definition used above i.e two viral load results ≥1000 copies/ml instead of least two high  viral load results. | 109-110 |  |
|  | I suggest that Categorization and coding of independent variables Table 1 is not necessarily needed because these are standard or common variables or table 1 can be included as supplementary material. | 123 |  |
| Data analysis plan | It looks like objective 1 analysis is not described because Objective 2 and 3 are specifically mentioned. Also, the authors may consider a brief mention of what was the objective1, 2 and 3 after the words ‘**’For objective 2...**  Example: for objective 2 (time to attrition)…” |  |  |
| **Results** |  |  |  |
| Socio-demographic and clinical characteristics | Improve sub title to read ***‘’study participants characteristics***’’ | 149 |  |
|  | There is inconsistence use of the viral load results description. Line 159  unsuppressed (≥1000 copies/mL) while earlier the authors used “high viral load” and “inconsistent suppression.” | 159 | Improve consistency throughout the manuscript. |
|  | Table 1 label is repeated and, no units for the following variables-age, duration on ART, CD 4 and VL results. Correct the typo error **CD4 4-at baseline.**  Define first and second line regimens as foot notes  Zone, COMMUNITY and OTHERS do not need capital letters. | 161 |  |
|  | It is unclear why for years 2–4 the authors provided point estimates only but no confidence intervals. | 170-171 |  |
|  | Correct the typo error Figure 4.3 | 176 |  |
|  | The following sentence need revision **‘’To estimate attrition from HIV care among adolescent girls and young women (AGYW) over a five-year follow-up period, survival analysis revealed a progressive decline in retention over time’'**  ’’ Survival analysis over the five‑year follow‑up period showed a progressive decline in retention among AGYW. | 195-196 |  |
| Discussion | The authors did not mention what potential reasons may explain lower attrition rates reported in Kenya, Cameroon, and Mozambique than the current study | 236-238 | Briefly state a plausible reason/s for differences. |
|  | This sentence ‘’Marital status influenced retention, with married and divorced AGYW experiencing higher attrition than single peers’’ need brief description/ potential reasons why findings vary across settings. The authors could add a brief contrast, where in some contexts marriage has been associated with improved support and retention. Also, consider whether your cohort’s had a fewer married AGWY (22% vs single 76%) and more young adults which may partially explain the association. | 247-248 |  |
|  | It is unclear what the authors mean for current study in Tanzania ‘’Clients on first-line ART had higher attrition than those on second-line regimens, likely due to the enhanced monitoring and support often provided to second-line clients. The reference provided in from Rwandan study. In this study of adults aged 15 years and above, the age group 15-29 years were 19% out of 1688. | 249-250 | **Note:** Tanzania national guideline does not segregate guidance on routine provision of enhanced adherence counselling and support to PLHIV receiving ART. |
|  | The contradiction mentioned in discussion on attrition decreased with advanced WHO clinical stages, possibly due to more intensive care for those with  severe disease in Tanzania contradicts with which other studies?  The reference is only one study from rural Mozambique where participants were adults 15 years and above and, AGWY 15-24 years were 14.2% out of 16321 all adults. | 251-252 | Please revise for clarity by comparing the age group 15-24 years. |
|  | The following finding is not discussed. Entry point into care also impacted outcomes; AGYW diagnosed through PMTCT/RCH and OPD  services had higher attrition compared to those entering via VCT or community channels. | 253-254 | Please |
| **Recommendations** | The recommendations provided in from lines 280-290 where are they from? Is it from other studies in similar settings? Some are already existing in Tanzania. The authors need to clarify what strategies are already in place but may be sub optimal but should be supported by the current study findings. Recommending new strategies need reference and describe briefly what findings relate or support what is recommended. | 280-290 |  |
| **Conclusion** | Specify ‘’first-year post-enrolment of ..ART? or in HIV care? | 303 | Specify which enrolment (ART initiation or HIV care). These two categories have different meanings in HIV national program. |
|  | After conclusion there are good recommendations supported by study findings. | 304-310 | The authors need to revise the recommendations to avoid repetition of recommendations. |
