# Supplementary material for: Trends of attrition from HIV care and its predictors among adolescent girls and young women with inconsistent viral load suppression results in Mainland Tanzania, 2016–2024
Source: PLOS Glob Public Health. 2026 Feb 11;6(2):e0005489. doi: 10.1371/journal.pgph.0005489 (PMC12893582; doi:10.1371/journal.pgph.0005489)
Supplement: Attachment — Submitted filename: Response to Reviewers.docx [file pgph.0005489.s003.docx]

**PGPH-D-25-03362: DECISION REVISION REQUIRED**

**COVER LETTER ON HOW EDITOR’S AND REVIWERS’ COMMENTS WERE ADDRESSED**

Date: 19^th^ December, 2025

**Authors**: Anthony Charles Kavindi¹*, Jegede Feyisayo Ebeneezer^4^, Asteria Karungi Nyongoli¹, Deogratius W. Kinoko^1^, Laura J. Shirima¹, Festo Charles^3^, Marion Sumari-de Boer^1,2,5,6^

**Affiliation of Authors**:

^1^School of Public Health, KCMC University, P.O. Box 2240, Moshi, Tanzania.

^2^Kilimanjaro Clinical Research Institute, Moshi, Tanzania.

^3^Management and Development for Health (MDH), Dar es Salaam, Tanzania.

^4^ C/o Bayero University Kano, Department of Life Science, Kano, Nigeria.

^5^Amsterdam Institute for Global Health and Development, Amsterdam, the Netherlands.

^6^ Kilimanjaro Christian Medical Centre, Moshi, Tanzania.

*Corresponding author: Anthony Charles Kavindi

E-mail address: * [kavindianthony@gmail.com](mailto:kavindianthony@gmail.com)

**Addressing the Editor-in-Chief Comments of Reviewers**

I am enclosing herewith response to editor-in-chief and individual reviewer comments, concerns and suggestion on the manuscript entitled **“Trends of attrition from HIV care and its predictors among Adolescent Girls and Young Women with inconsistent viral load suppression results in Mainland Tanzania, 2016–2024”.** Effort made to address all comments, concerns or suggestions are presented below for the editor-in-chief and reviewers (n=2). We have provided responses to all Reviewer comments (n=4) and Reviewer 2 comments (n=3) respectively.

**REVIEWERS’ AND EDITOR’S COMMENTS**

| **Comment** | **Author Response** |
| --- | --- |
| **Editor Comment** | |
| 1.General comment about the manuscript | We thank both editor-in-chief and two reviewers for the constructive and insightful comments and feedback. We have revised the manuscript accordingly in line with reviewers’ comments and suggestions to improve clarity, methodological description, objectives alignment, analysis, and outcomes. Specifically, we have provided a detailed response to each comment, concerns and suggestions effect same in the manuscript tracked changes revised manuscript accordingly. |
|  |  |
| **Reviewers Comments (1, 2):** |  |

**REVIEWER 1**

| **Comment** | **Author Response** |
| --- | --- |
| 1. Comment 1: “Methodology: study design & datasource, heading overlap with data source and extraction. You may consider differentiating the study design+population from the data source+data extraction to improve clarity and avoid repetition.”  2. Comment 2: “The covariates are presented in a table. Consider adding a concise paragraph describing key variables and their categorization. Perhaps detailed coding and operational definitions should be presented in a supplementary table.”    3. Comment 3: “The analysis plan refers to Objective 2 and Objective 3; however, the study objectives are not stated anywhere earlier in the manuscript. They should be explicitly stated to improve coherence.”  4. Comment 4: “Also, this contradicts ‘shared frailty at the individual level’. Please clarify the intended clustering structure. Did you model with the shared or individual-level frailty?” | Response1.Thank you for this observation and suggestion. We have separated the study design and study population from the data source and data Extraction into two distinct sub-sections to avoid repetition. The Study design and population section now clearly describes a retrospective cohort design, target population (AGYW aged 15–24 years), study period (2016–2024), and inclusion/exclusion criteria. The data source and extraction section now specifically focuses on the CTC2 macro database, variables extraction, cleaning procedures, and quality control steps. We believe that this separation eliminates redundancy and improves readability.  Response2: We agree with reviewer on covariates variables description and supplementary table suggestion. and have revised the manuscript accordingly. A descriptive paragraph summarizing the main independent variables, rationale, and categorical groupings have been added. Additionally, Supplementary Table S1 provides detailed coding schemes and operational definitions for all variables, including HIV viral load thresholds, definitions of inconsistent viral suppression, and retention/attrition definitions.  Response3: The authors appreciate the reviewer insights on improving objective conference comment. We have added a list of study objectives clearly stated at the end of the Introduction section. These objectives now align precisely with the descriptive, survival, and multivariable regression analyses presented in the Methods and Results sections.  Response 4: The authors appreciate the reviewer for pointing out this important methodological gap and clarification. The intended modelling approach was shared in the frailty at the individual level, not facility-level frailty. A shared frailty Cox proportional hazards model with individual-level frailty was used to account for unobserved heterogeneity between repeated measurements within the same individual. This approach we believe adjusted for intra-individual correlation and provides more accurate estimates of hazard ratios for predictors of attrition while acknowledging that each participant may have unique risk factors not fully captured by observed covariates. |

**REVIEWER 2**

| **Comment** | **Author Response** |
| --- | --- |
| Comment 1: “The manuscript would benefit from clearer operational definitions of key outcomes, particularly inconsistent viral load suppression.”  Comment 2: “Further discussion linking the findings to existing regional and global literature, as well as clearer description of programmatic and policy implications, would strengthen the paper.”  Comment 3: “I recommend minor revisions to improve clarity and consistency.” | 1.Response: The authors appreciate the reviewer for this valuable suggestion. We have strengthened the operational definitions section by providing precise definitions of persistent unsuppressed HIV viral load, inconsistent HIV viral load suppression, and attrition from ART care. Additionally, we have clarified the HIV viral load threshold (<1,000 copies/mL per as per the Tanzania national ART guidelines) and specified time windows thresholds for first and repeat or follow-up HIV viral load testing. These definitions are further detailed in Supplementary Table S1.  **Response 2**: The reviewer comment on linking findings to current regional and global literature landscape and highlighting future policy implication is well received. We have expanded the Discussion section to include studies comparisons from other Africa settings such as Eastern and Southern Africa, including Kenya, Uganda, and South Africa, focusing on AGYW retention and HIV viral suppression challenges. Further, we also highlighted structural and contextual barriers such as stigma, mobility, and socio-economic vulnerability. The study findings broader implication and policy implication have been strengthened by recommending age-tailored adherence counseling, enhanced facility-level tracking systems, early identification of unsuppressed AGYW, and differentiated service delivery models.  **Response 3:** We conducted a thorough revision of the manuscript to improve clarity and consistency. Minor grammatical errors, formatting, and structural adjustments were made where necessary. |

In conclusion, the authors would like to sincerely appreciate the editor-in-chief and both reviewers for those useful constructive insightful comments, suggestions and feedback that have significantly raised the work quality, improve clarity and better flow for good readability of the manuscript during the review process.

We hope all comments, concerns and suggestions have been sufficiently addressed to meet *PLOS GLOBAL PUBLIC* journal acceptable criteria for publication.

Sincerely,

Anthony Charles Kavindi on behalf of all the authors.

KCMC University

19^th^ December 2025
