# Supplementary material for: Trends of attrition from HIV care and its predictors among adolescent girls and young women with inconsistent viral load suppression results in Mainland Tanzania, 2016–2024
Source: PLOS Glob Public Health. 2026 Feb 11;6(2):e0005489. doi: 10.1371/journal.pgph.0005489 (PMC12893582; doi:10.1371/journal.pgph.0005489)
Supplement: Attachment — Submitted filename: PONE Review 02012026.docx [file pgph.0005489.s004.docx]

**PONE Review: PGPH-D-25-03362**

**Title:** Trends of attrition from HIV care and its predictors among Adolescent Girls and Young Women with inconsistent viral load suppression results in Mainland Tanzania, 2016–2024

**General review of the revised manuscript**

I have reviewed the revised manuscript and found that the authors have addressed all the comments; however, a few minor edits are still required before publication. Please see the table below.

| **Section** | **Comment** | **Line number** |
| --- | --- | --- |
| Introduction | Correct spelling UANIDS | 58 |
| Methods | It is essential that the term inconsistent viral load suppression needs to be clearly defined in the manuscript.  Note that two viral load results ≥1000 copies/ml at least six months after ART initiation is the standard definition for non-viral suppression or ART virological failure.  Ideally, inconsistent viral load suppression would have been a situation whereby PLHIV achieves an undetectable viral load at least 6 months after ART initiation, but subsequently experience detectable viral load result/s ≥1000 copies/ml.  To avoid confusion, refer to this sentence in conclusion: ***Notably, individuals with an initial unsuppressed viral load were more likely to disengage from*** ***care.*** |  |
| Results | Table 1: First line and second line not defined. Not every reader knows what Tanzanian ART regimens. | 170-171 |
| Discussion | A citation is not inserted | 253 |
|  | Correct the word **form** to read **from** | 254 |
