# Supplementary material for: Trends of attrition from HIV care and its predictors among adolescent girls and young women with inconsistent viral load suppression results in Mainland Tanzania, 2016–2024
Source: PLOS Glob Public Health. 2026 Feb 11;6(2):e0005489. doi: 10.1371/journal.pgph.0005489 (PMC12893582; doi:10.1371/journal.pgph.0005489)
Supplement: Attachment — Submitted filename: Response_to_Reviewers_auresp_2.docx [file pgph.0005489.s005.docx]

**PGPH-D-25-03362: DECISION REVISION REQUIRED**

**COVER LETTER ON HOW EDITOR’S AND REVIWERS’ COMMENTS WERE ADDRESSED**

Date: 16^th^ January, 2026

**Authors**: Anthony Charles Kavindi¹*, Jegede Feyisayo Ebeneezer^4^, Asteria Karungi Nyongoli¹, Nagalal William^2^, Deogratius W. Kinoko^1^, Laura J. Shirima¹, Festo Charles^3^, Marion Sumari-de Boer^1,2,5,6^

**Affiliation of Authors**:

^1^School of Public Health, KCMC University, P.O. Box 2240, Moshi, Tanzania.

^2^Kilimanjaro Clinical Research Institute, Moshi, Tanzania.

^3^Management and Development for Health (MDH), Dar es Salaam, Tanzania.

^4^ C/o Bayero University Kano, Department of Life Science, Kano, Nigeria.

^5^Amsterdam Institute for Global Health and Development, Amsterdam, the Netherlands.

^6^ Kilimanjaro Christian Medical Centre, Moshi, Tanzania.

*Corresponding author: Anthony Charles Kavindi

E-mail address: * [kavindianthony@gmail.com](mailto:kavindianthony@gmail.com)

**Addressing the Editor-in-Chief Comments of Reviewers**

I am enclosing herewith response to editor-in-chief and individual reviewer comments, concerns and suggestion on the manuscript entitled **“Trends of attrition from HIV care and its predictors among Adolescent Girls and Young Women with inconsistent viral load suppression results in Mainland Tanzania, 2016–2024”.** Effort made to address all comments, concerns or suggestions are presented below for the editor-in-chief and reviewers (n=2).

**REVIEWERS’ AND EDITOR’S COMMENTS**

| **Section** | **Comment** | **Line number** | **Author Response** |
| --- | --- | --- | --- |
| Introduction | Correct spelling UANIDS | 58 | The spelling of *UNAIDS* has been corrected (previously misspelled as UANIDS). |
| Methods | It is essential that the term inconsistent viral load suppression needs to be clearly defined in the manuscript.  Note that two viral load results ≥1000 copies/ml at least six months after ART initiation is the standard definition for non-viral suppression or ART virological failure.  Ideally, inconsistent viral load suppression would have been a situation whereby PLHIV achieves an undetectable viral load at least 6 months after ART initiation, but subsequently experience detectable viral load result/s ≥1000 copies/ml.  To avoid confusion, refer to this sentence in conclusion: ***Notably, individuals with an initial unsuppressed viral load were more likely to disengage from*** ***care.*** |  | We agree that a clear definition is essential to avoid confusion. We have now explicitly defined inconsistent viral load suppression in the Methods section. The revised text clarifies that, inconsistent viral load suppression in this study refers to individuals who initially achieved viral suppression (undetectable viral load ≥6 months after ART initiation) but subsequently experienced one or more detectable viral load results ≥1000 copies/ml during follow-up. |
| Results | Table 1: First line and second line not defined. Not every reader knows what Tanzanian ART regimens. | 170-171 | We have revised Table 1 to clearly define first-line and second-line ART regimens. Full drug names have been added in the table footnote to ensure clarity for readers unfamiliar with Tanzanian ART regimens. |
| Discussion | A citation is not inserted | 253 | The missing citation has now been inserted in the Discussion section. |
|  | Correct the word **form** to read **from** | 254 | We believe these revisions have addressed all remaining concerns and improved the clarity and consistency of the manuscript. We are grateful for the reviewer’s constructive feedback, which has strengthened the final version of the paper. |

In conclusion, the authors would like to sincerely appreciate the editor-in-chief and both reviewers for those useful constructive insightful comments, suggestions and feedback that have significantly raised the work quality, improve clarity and better flow for good readability of the manuscript during the review process.

We hope all comments, concerns and suggestions have been sufficiently addressed to meet *PLOS GLOBAL PUBLIC* journal acceptable criteria for publication.

Sincerely,

Anthony Charles Kavindi on behalf of all the authors.

KCMC University

16^th^ January, 2026
